# Application of low-intensity pulsed therapeutic ultrasound on mesenchymal precursors does not affect their cell properties

**DOI:** 10.1371/journal.pone.0246261

**Published:** 2021-02-11

**Authors:** Beatriz de Lucas, Laura M. Pérez, Aurora Bernal, Beatriz G. Gálvez

**Affiliations:** 1 Faculty of Biomedical and Health Sciences, Universidad Europea de Madrid, Madrid, Spain; 2 Centro Nacional de Investigaciones Cardiovasculares (CNIC), Madrid, Spain; University of Zaragoza, SPAIN

## Abstract

Ultrasound is considered a safe and non-invasive tool in regenerative medicine and has been used in the clinic for more than twenty years for applications in bone healing after the approval of the *Exogen* device, also known as low-intensity pulsed ultrasound (LIPUS). Beyond its effects on bone health, LIPUS has also been investigated for wound healing of soft tissues, with positive results for various cell processes including cell proliferation, migration and angiogenesis. As LIPUS has the potential to treat chronic skin wounds, we sought to evaluate the effects produced by a conventional therapeutic ultrasound device at low intensities (also considered LIPUS) on the migration capacity of mouse and human skin mesenchymal precursors (s-MPs). Cells were stimulated for 3 days (20 minutes per day) using a traditional ultrasound device with the following parameters: 100 mW/cm^2^ with 20% duty cycle and frequency of 3 MHz. At the parameters used, ultrasound failed to affect s-MP proliferation, with no evident changes in morphology or cell groupings, and no changes at the cytoskeletal level. Further, the migration and invasion ability of s-MPs were unaffected by the ultrasound protocol, and no major changes were detected in the gene/protein expression of ROCK1, integrin β1, laminin β1, type I collagen and transforming growth factor β1. Finally, RNA-seq analysis revealed that only 10 genes were differentially expressed after ultrasound stimulation. Among them, 5 encode for small nuclear RNAs and 2 encode for proteins belonging to the nuclear pore complex. Considering the results overall, while the viability of s-MPs was not affected by ultrasound stimulation and no changes were detected in proliferation/migration, RNA-seq analysis would suggest that s-MPs do respond to ultrasound. The use of 100 mW/cm^2^ intensity or conventional therapeutic ultrasound devices might not be optimal for the stimulation the properties of cell populations. Future studies should investigate the potential application of ultrasound using variations of the tested parameters.

## Introduction

Cell fate can be regulated by their relationship with other cells and by chemical and physical factors [1]. Cells are able to sense environmental mechanical signals and respond them by translating into biochemical responses. This process is known as mechanotransduction. Mechanical wave are sense by cell mechanoreceptors, that are transmembrane proteins that link with the cytoskeleton [2]. Once the mechanoreceptor receives the mechanical stimuli lead to the activation of different signaling pathways with finally rule cell fate, including quiescence, cell adhesion, proliferation, migration, differentiation, or apoptosis [3]. Hence, ultrasound, as mechanical stimuli, has the potential to regulate cell behavior.

Ultrasound has been widely used in biomedicine for more than fifty years—both as a safe and non-invasive diagnostic tool for real-time imaging (no radiation is emitted) and, more recently, as a surgical or therapeutic modality for various disorders or diseases (bone fractures, cancer and kidney stone ablation, among others) [4–6]. The breadth of practical applications of ultrasound waves are due, in part, to the ability to alter their properties depending on the parameters chosen. Indeed, new applications for ultrasound are continually evolving not only in the biomedical field but also in others such as the food industry [7]. Ultrasound can be used also for improve stem cell delivery and survival due to offers real-time guidance. Nowadays multimodal approaches are being developed in combination with photoacoustic imaging and magnetic particle imaging [8,9].

Low-intensity pulsed ultrasound (LIPUS), a form of ultrasound transmitted transcutaneously as high frequency acoustic pressure waves, has been approved for over two decades for bone fracture healing [10–12]. Not only has been continued the study their effects on osteogenic/bone repair [13,14], but also its study has been extended to new areas. Its use in soft tissue regeneration has drawn attention to potentially important novel properties including the stimulation of cell proliferation [15,16], migration [17,18] and angiogenesis [19,20]. Three major parameters for LIPUS are intensity, frequency and exposure time: typical parameters used are an ultrasound carrier frequency of 1.5 MHz, power intensity of 30 mW/cm^2^ and daily duration of 20 min. Nevertheless, a range of intensity levels has been reported *in vitro*, from 0.03 to 0.1 W/cm^2^ SATA (spatial average-temporal average) [21]. Using these parameters, the heat and cavitation risk is considered negligible [22]. Most if not all physiotherapy clinics have a conventional therapeutic ultrasound device, whereas a LIPUS device, for example the Exogen 2000+ (Smith & Nephew Inc.), is less common and considerably more expensive. Accordingly, the use the lower intensities of conventional therapeutic ultrasound devices might be an economical alternative to obtain LIPUS-like effects.

Mesenchymal precursors (MPs) are adult stem cells present in virtually every organ and are characterized by their ability to differentiate into various mesenchymal cell lineages. They are relatively easy to obtain from many cell depots and are endowed with important therapeutic properties including immunomodulatory potential and secretory and migratory functions [23–25]. Accordingly, they have a high potential to be used in regenerative/reparative processes. However, a limitation for their use as a therapy is their propensity to remain at the site of injection and their lack of migration to sites of damage, which drives the development of new strategies to overcome these challenges [26].

Due to the great potential of LIPUS for chronic skin wound healing, activation of fibroblast proliferation and migration [27], and production of extracellular components [28] such as collagen [29,30], we aimed to study whether ultrasound waves generated by a conventional device, at the lowest intensity setting, could reproduce these functions in skin mesenchymal precursors (s-MPs).

## Material and methods

### Isolation and expansion of mesenchymal precursors

Mice were maintained and used in accordance with the National Institutes of Health Animal Care and Use Committee. Protocols were approved by the Research Ethics Committee (CEI) of CNIC (Centro Nacional de Investigaciones Cardiovasculares). Mice were sacrificed by CO2 chamber. Human samples skin explants were obtained after esthetic ear surgery conformed to the principles set out in the WMA Declaration of Helsinki and the NIH Belmont Report. Protocols were approved by the Health Sciences Research Committee of Universidad Europea de Madrid with reference number CIPI/069/17. The isolation of s-MPs was performed using the explant technique described previously [31]. Skin explants from adult mice and adult humans were collected and dissected into 1–2 mm pieces. The tissue explants were placed in the center of 24-plate wells with the well contours coated with Matrigel ^™^ (BD Biosciences, Franklin Lakes, NJ, USA). Explant culture medium, referred to as complete medium, consisted of Dulbecco’s modified Eagles’s medium (DMEM) supplemented with 10% fetal bovine serum (both from Sigma-Aldrich, St Louis, MO, USA), 105 U/mL penicillin/streptomycin, 2 mM L-glutamine and 10 mM Hepes (all from Lonza, Basel, Switzerland). Cultures were maintained for several days at 5% CO_2_/95% air atmosphere at 37°C and, after 1 week, cells appeared around the explant. Cell expansion was performed on gelatin-free culture plates. Studies were performed using cells from passage 10 to 20. Cells were characterized by flow cytometry as follows: human cell populations were positive for CD105 and negative for CD34; murine cell populations showed a variable expression of CD34 and Sca-1. Both populations were positive for CD44 and negative for CD31 and CD45, confirming that they are non-hematopoietic mesenchymal precursors. The experiments were done with two independent cell populations from both human (H1 and H2) and mouse (M1 and M2) samples. However, for RNA-sequencing we used four human cell populations, to strengthen the biological interpretation of the results.

### Ultrasound application (low-intensity pulsed therapeutic ultrasound)

**Fig 1:**
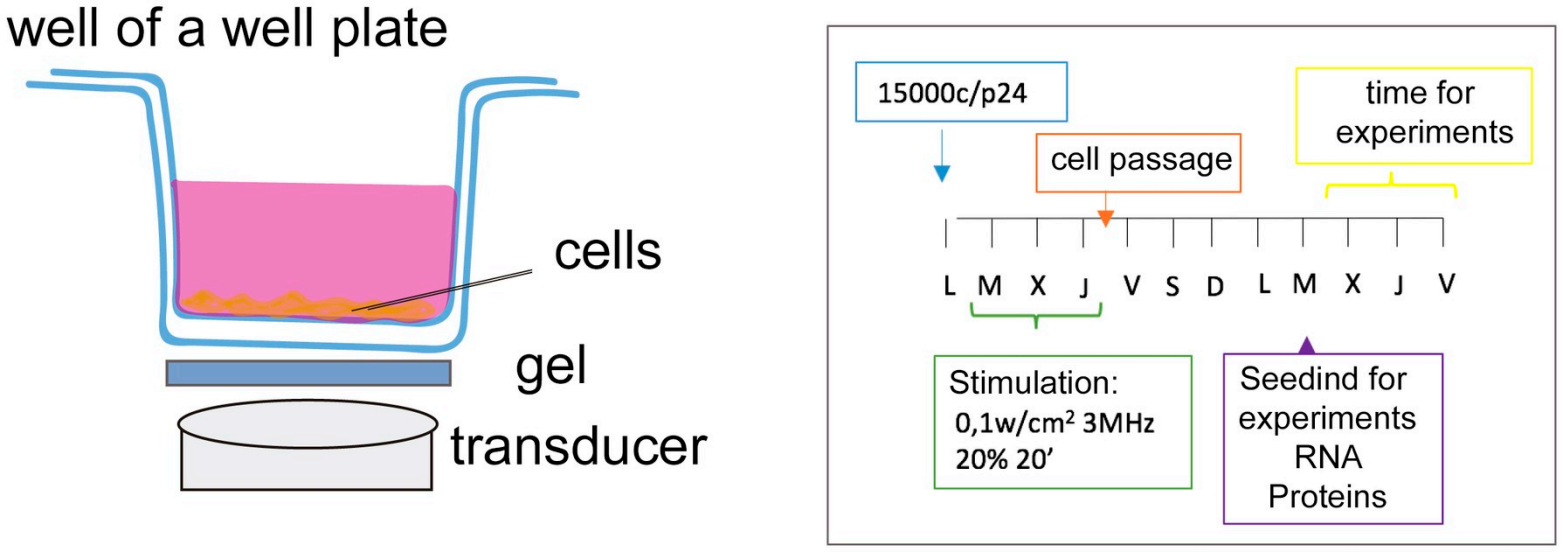
Scheme of the ultrasound application and the experimental design. The application of LIPUS was performed with a gel between the transducer and the bottom of the plate where the cells are attached. The experiments were conducted 5 days after the final application of LIPUS. Ultrasound attenuation within a polystyrene standard culture plate (1.2 mm) has been described as not significant (less than 0.3 dB or 4% over the frequency range from 1 to 3 MHz) [32].

Application of ultrasound (therapeutic LIPUS) to cells was performed using the Medisound 3000 device (Globus, Codognè, Italy), which is approved by the EU for use in hospitals and physiotherapy clinics. A total of 1.5×10^4^ cells were seeded in each well of a 24-well plate and were maintained for 24 h in the humidified incubator before stimulation. Application of LIPUS to s-MPs was performed using the following parameters (Fig 1): 100 mW/cm^2^ intensity (lowest available power) and 3 MHz frequency. The LIPUS protocol consisted of 20 minutes each day with an ultrasound pulsed at 20% (1:4) at 1000 Hz, for three consecutive days. LIPUS was applied outside of the incubator at room temperature, with control cultures treated identically (without LIPUS). Once the daily application was completed, the cells were returned to the incubator. On day four cells were trypsinized and expanded to ~1×10^6^ cells for experiments. All experiments were carried out five days after the final LIPUS application, which was necessary to expand the cells (Fig 1).

### Proliferation assay–cell counting

For cell counting experiments, 1×10^4^ cells were seeded in each well of a 24-well plate and counted over three days. The results were expressed as a proliferation curve representing the total number of cell present on the plate each day.

### Proliferation assay–bromodeoxyuridine incorporation

The bromodeoxyuridine (BrdU) assay (Merck KGaA, Darmstadt, Germany) was used for proliferation analysis, which is based on the incorporation of the thymidine analog BrdU into DNA strands during replication. Briefly, 5×10^3^ cells were seeded in each well of a 96-well plate. The next day, BrdU (1:2000 dilution) was added to the culture and cells were maintained for 24 h. Cells were then fixed and washed and incorporated BrdU was detected with an anti-BrdU antibody (1:100, 1 h incubation at room temperature), which was visualized with an HRP-conjugated secondary antibody (1:1000, 30 min at room temperature). Finally, after washing, the chromogen substrate was added for 30 min in the dark for the development of the peroxidase reaction. Once the STOP solution was added, the optical density was read in a spectrophotometer (SPECTROstar^Nano^; BMG LABTECH, Aylesbury, UK) at 450 nm.

### Fluorescence microscopy

Cells (2.5×10^4^) were plated on 0.1% gelatin-coated coverslips in a 24-well plate and were maintained in culture for 24 h. Then, coverslips were washed with phosphate buffered saline (PBS), fixed with 4% paraformaldehyde for 15 min, and blocked with 1% goat serum for 1 h at room temperature. Primary antibodies used were rabbit anti-tubulin (1:100 concentration; Abcam, Cambridge, UK) and phalloidin-TRITC (1:100 dilution; Sigma-Aldrich). Antibody staining was carried out in antibody dilution buffer overnight at 4°C. For tubulin staining, after washing with PBS, a goat anti-rabbit Alexa488 secondary antibody was added (1:400 dilution; Invitrogen, Life Technologies, Carlsbad, CA, USA) for 1 h in the dark at room temperature. Coverslips were co-stained with DAPI (300 nM; Sigma-Aldrich) for 10 min at room temperature and mounted with ProLong Antifade reagent (Invitrogen) on glass slides. Images were observed with a Leica DM2000 LED (Leica Microsystems, Wetzlar, Germany).

### Wound-healing assay

To evaluate collective migration, we used the wound-healing assay. Confluent s-MP cultures were scratch-wounded with a sterile micropipette tip, washed with PBS to remove cellular debris, and replenished with complete medium. Cells were maintained in culture and images were captured at different times using a Motic AE31 microscope (Motic, Hong Kong, China). The calculation of the wound area was performed with ImageJ software (Bethesda, MD, USA). The results were expressed as a percentage of wound closure.

### Transwell migration assay

To evaluate individual migration, we used Transwell chambers (Corning Inc., MA, USA) with 6.5 mm-diameter permeable membranes and 8-μm pore size filters. Murine (2×10^4^) and human (5×10^4^) s-MPs were plated in 80 μl of medium in the upper chamber of the Transwell chamber (placed on 24-well plates) and complete culture medium was placed in the lower chamber. After 24 h, chambers were fixed with 4% glutaraldehyde for 2 h and then stained overnight with 1% toluidine blue (both from Sigma-Aldrich). Cells on the lower side of the membrane were visualized with a Motic AE31 microscope and counted in five randomly-selected 10× fields using ImageJ software (Bethesda, MD, USA). The results were expressed as migrated cells per field.

### Transwell invasion assay

Invasion assays were performed following the same protocol as the Transwell migration assays, but membranes were coated beforehand with 1% gelatin in PBS for 1 h at 37°C.

### Quantitative PCR

Total RNA was extracted from s-MPs using the Easy-spin Total RNA Extraction Kit (iNtRON Biotechnology, Sangdaewon-Dong, South Korea) and its concentration was quantified in a spectrophotometer (ND1000 NanoDrop, Thermofisher Scientific, Rockford, IL, USA). RNA was reverse-transcribed to cDNA using PrimeScript^™^ RT Master Mix (TAKARA Bio. Inc., Kusatsu, Japan). Quantitative PCR (qPCR) was performed using SYBER^®^ Green PCR Master Mix (Premix Ex Taq^™^, TAKARA Bio. Inc.) on the CFX96 Touch Deep Well^™^ Real-Time PCR Detection System (Bio-Rad Laboratories, Richmond, CA, USA). Thermal cycling parameters were as follows: first step of 94°C for 10 min, then 40 cycles of 94°C for 15 s and the primer-specific annealing temperature for 1 min (56°C). The last step was the melting curve analysis. qPCR was performed using the primers in Table 1.

**Table 1:** Primers for qPCR.

Gene name	Forward	Reverse
*GAPDH*	AATGCATCCTGCACCACCAA	GTGGCAGTGATGGCATGGAC
*ROCK1*	TGCCATGTTAAGTGCCACAG	AGGGGAAGCACGAACAAAAC
*COL1A1*	TGATGGGATTCCCTGGACCT	TCCAGCCTCTCCATCTTTGC
*TGFB1*	CTGCTGACCCCCACTGATAC	GTGAGCGCTGAATCGAAAGC
*LAMB1*	AGGAGACTGGGAGGTGTCTC	GTCAGAGCCGTTACAGTGCT
*ITGB1*	GCCGCGCGGAAAAGATG	TGAATTTGTGCACCACCCAC

### Western blotting

Cell lysates were extracted and lysed directly on ice using Laemmli buffer. Lysates were resolved by electrophoresis using 12% SDS-PAGE and proteins were transferred to nitrocellulose membranes for immunodetection. Membranes were blocked with 5% nonfat milk in PBS for 1 h at room temperature and subsequently incubated overnight at 4°C with a 1:1000 dilution of the primary antibody (β1laminin, β1integrin, βactin; Abcam, Cambridge, UK), followed by a quick wash in PBS containing 0.1% Tween-20 and detection with the appropriate secondary antibody (anti-rabbit HRP). Blots were visualized with the ECL reagent using a ChemiDoc XRS+ system (Bio-Rad Laboratories) and relative intensity was quantified by densitometry using ImageJ software (Bethesda, MD, USA).

### RNA-sequencing

RNA-sequencing library preparation and sequencing of the human cell samples was carried out by STABVida Lda (Caparica, Portugal). RNA integrity was checked on a Bioanalyzer 2100 (Agilent Technologies, Santa Clara, CA, USA). The Kapa Stranded Total RNA and Ribo-Zero Library Preparation Kit were employed for library construction, and sequencing was performed using the HiSeq 4000 Illumina Platform with 2×150 bp paired end reads. The bioinformatics analysis of the generated raw sequence data was carried out using CLC Genomics Workbench 11.0.1. Further quality control was performed by principal component analysis (PCA), hierarchical clustering (considering Manhattan distance), and heat map analysis. Differential expression was then calculated using multi-factorial statistical analysis based on a negative binomial model that used a generalized linear model approach influenced by the multi-factorial EdgeR method [33]. The differentially expressed genes were filtered using standard conditions [33], a p-value less than 0.05 and fold change over 2 or under -2. Raw data in fastq format are available with the accession number PRJNA662884 in the NCBI Biosample database (https://www.ncbi.nlm.nih.gov/sra/PRJNA662884).

### Data analysis

Statistical analysis and graphical representation of the results was performed using GraphPad Prism software (GraphPad Software Inc., San Diego, CA, USA). Values are expressed as mean ± standard deviation (SD) from 3 independent experiments. Data were checked for normality using the D’Agostino-Pearson test. Comparisons between groups were performed with one-way or two-way analysis of variance (ANOVA). The multiple comparisons test used for one-way ANOVA was Bonferroni’s and for two one-way ANOVA we used Tukey’s. Student’s t test was used when there was only one variable to consider. The specific analysis used is specified in the figure legends. Data was considered significantly different when P < 0.05; *P < 0.05; **P < 0.01; ***P < 0.001.

## Results

### Low-intensity ultrasound application procedure

**Fig 2:**
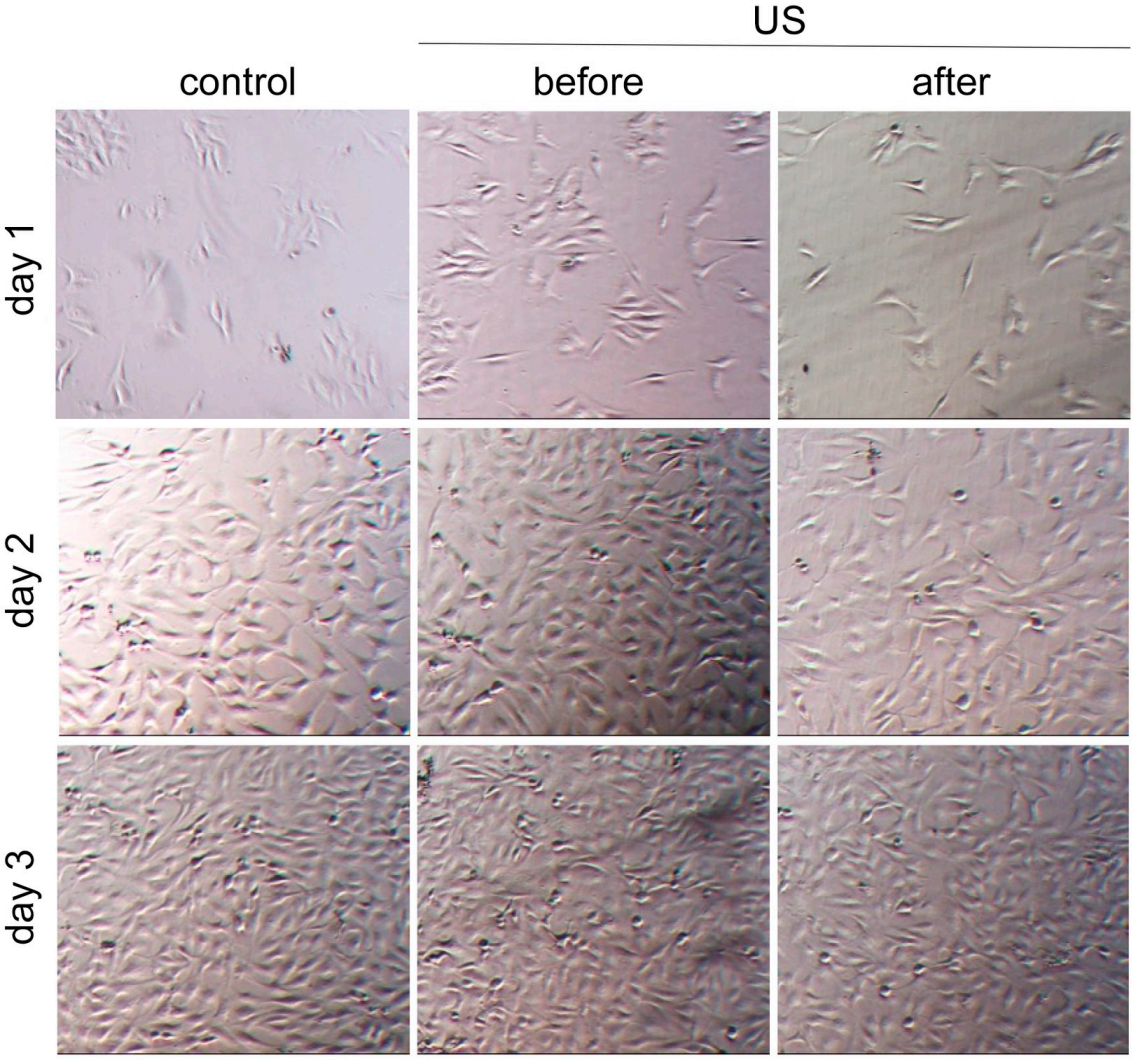
Application of LIPUS. Representatives phase contrast images of human s-MPs showing control cells and LIPUS-stimulated (US) cells before and after consecutive treatment during 3 days.

Ultrasound exposure (Medisound 3000) was performed on cells adhered to tissue culture plates, at low confluence (1.5×10^4^) and prior to experimental testing. We had previously discarded the application of ultrasound on suspended cells (as mechanotransduction occurs with adhered cells) or directly during experimental testing (ultrasound application would not be homogeneous). Attached cells were stimulated at 100 mW/cm^2^ with 3 MHz for 20 min, and a 20% duty-cycle during 3 days [17]. As shown in Fig 2, cells receiving the ultrasound application were morphologically indistinguishable from control cells. As a positive control of US stimulation, the increase of the intensity to 1 W/cm^2^ caused the death of the cells (S1 Fig).

### Low-intensity ultrasound stimulation does not produce noticeable changes in cytoskeleton organization

**Fig 3:**
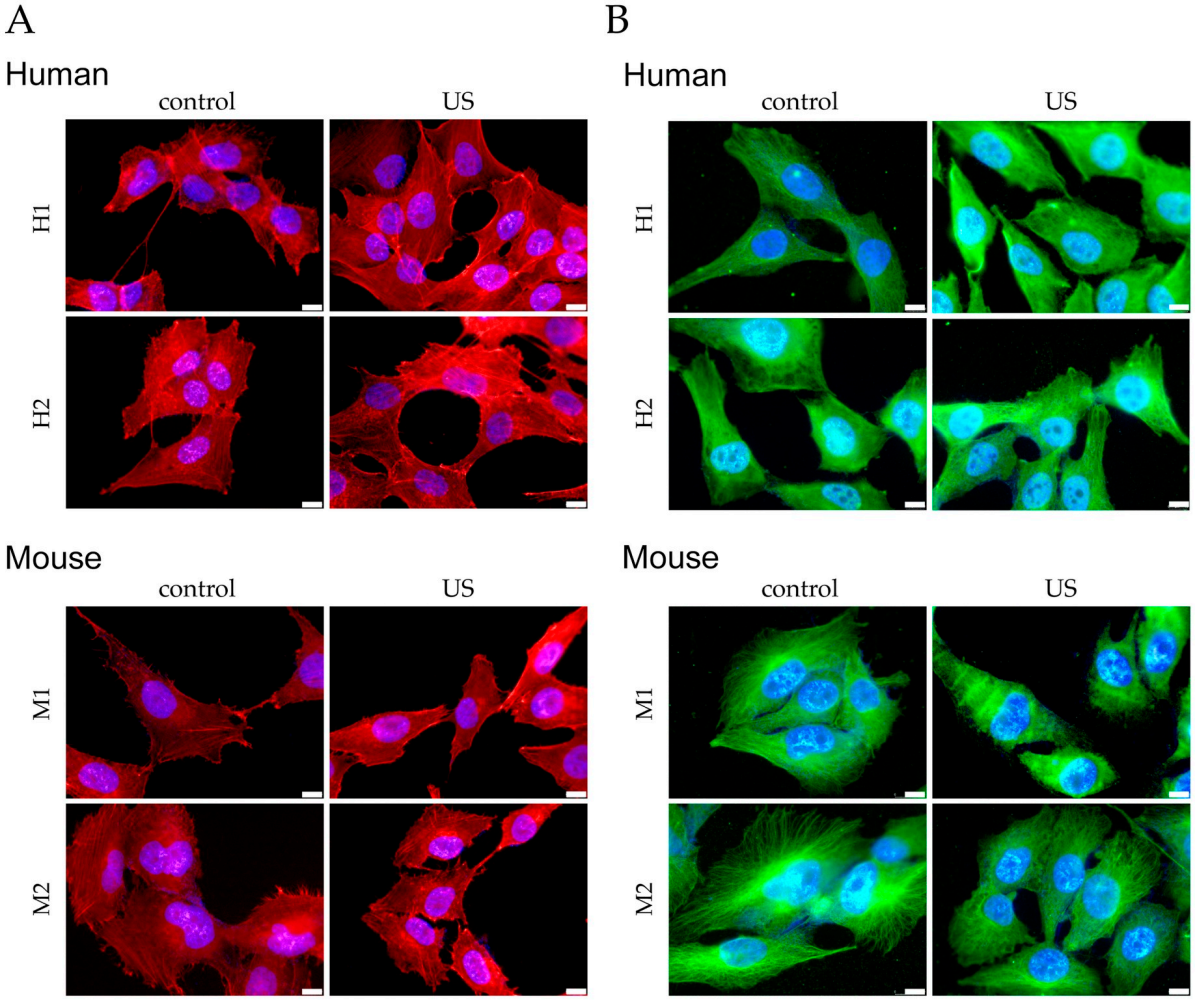
Application of LIPUS does not affect the cytoskeletal organization of s-MPs. Control cells and cells stimulated with LIPUS (US) for 3 days were stained with (A) phalloidin-TRITC to visualize F-actin (red) and (B) β-tubulin to visualize microtubules (green). Nuclei were counter-stained with DAPI (blue). Shown are two independent samples of mouse (M1 and M2) and human (H1 and H2) s-MPs. Images are shown at a magnification of 63×, scale bar 10 μm.

To assess whether the LIPUS stimulation protocol resulted in changes to the organization of the cytoskeleton in s-MPs, we analyzed the distribution of F-actin (actin filaments) and β-tubulin (microtubules) using fluorescence immunocytochemistry. Stimulated s-MPs from human and mouse had a comparable morphology to respective controls (Fig 3), with evident actin filaments and microtubules. No cytoskeletal reorganization could be detected (clusters or other structures) in the stimulated cells. Overall, the LIPUS protocol used with the selected parameters appears not to cause any manifest structural changes to cell morphology and the cytoskeleton.

### Low-intensity ultrasound stimulation does not affect cell proliferation

**Fig 4:**
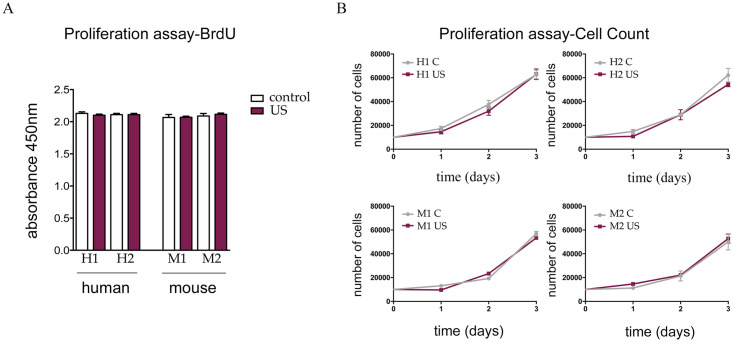
Application of LIPUS does not modify the proliferation capacity of s-MPs. (A) Cell proliferation was evaluated by BrdU incorporation. Data are shown from a representative experiment out of three performed and denote mean ± SD. No significant differences were found (Student’s t-test). (B) Cell proliferation was calculated by cell counting. Data are shown from a representative experiment out of three performed and denote mean ± SD. No significant differences were found (two-way ANOVA with Tukey’s multiple comparisons test). Shown are two independent samples of mouse (M1 and M2) and human (H1 and H2) s-MPs under control conditions or treated with ultrasound (US).

To question whether the LIPUS stimulation protocol impacted cell proliferative potential, we performed both BrdU incorporation and manual cell counting. The results of both assays indicated no differences in the proliferation rate of cells subjected to LIPUS stimulation when compared with control cells, with similar results for murine and human s-MPs (Fig 4).

### Low-intensity ultrasound stimulation does not affect migration capacity

**Fig 5:**
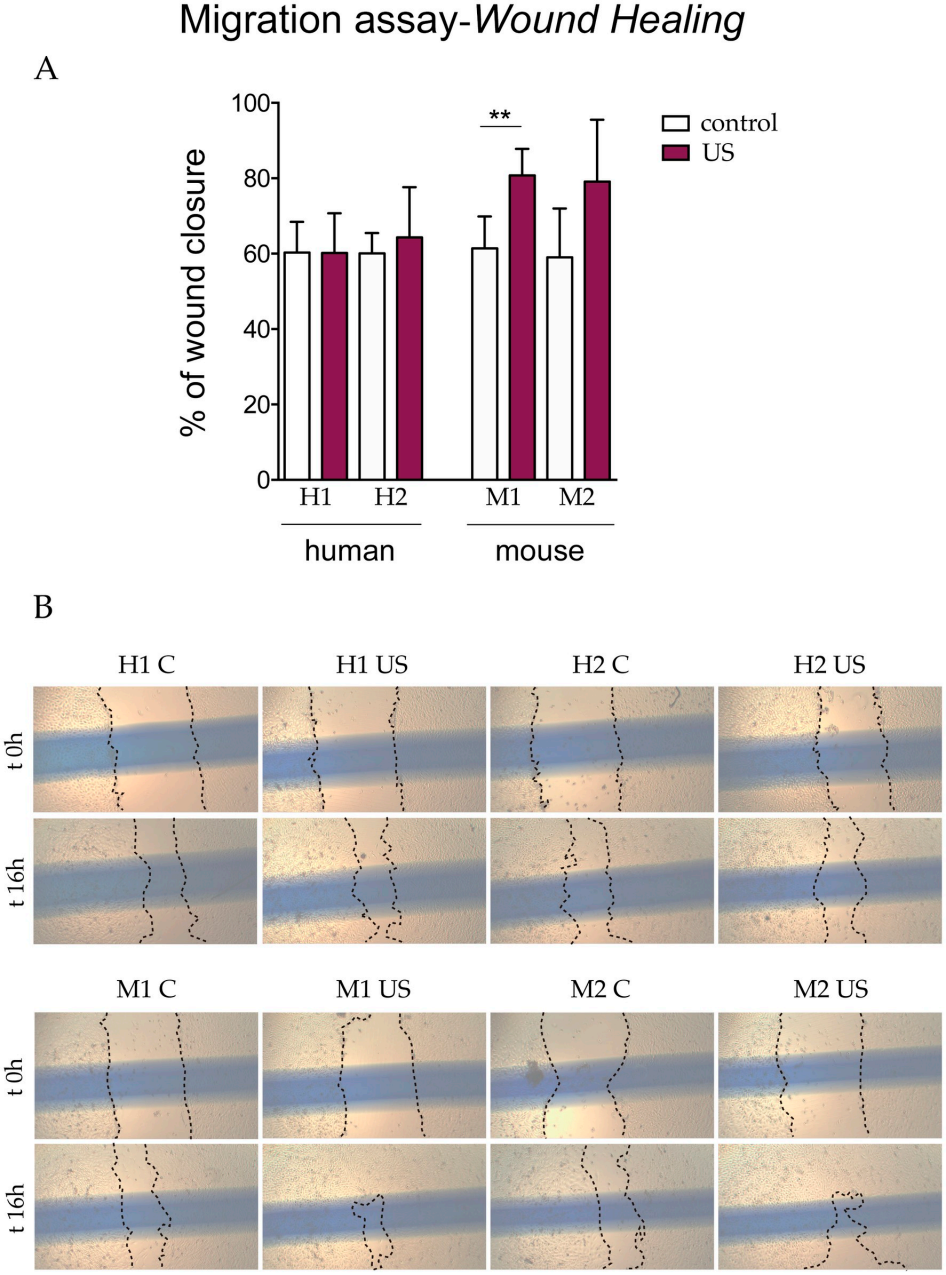
Wound healing migration assay. (A) Data are shown from a representative experiment out of five performed and denote mean ± SD (quantification at 16 h). Statistical analysis was performed using Student’s t test. **p < 0.01. (B) Representative images of the wound at time 0 h and 16 h after scratching. Shown are two independent samples of mouse (M1 and M2) and human (H1 and H2) s-MPs under control conditions or treated with ultrasound (US).

Next, to evaluate whether the LIPUS protocol impacted the capacity of s-MPs to migrate, an important attribute of MPs, we performed several migration-based assays. We first utilized the scratch wound-healing assay to study collective two-dimensional cell migration. No significant differences in wound closure were observed between human control and LIPUS-stimulated s-MPs (Fig 5). With respect to murine s-MPs, wound closure occurred faster in LIPUS-stimulated cells than in control cells, and this was significant for one of the two independent s-MP populations; however, the increase was modest (Fig 5).

**Fig 6:**
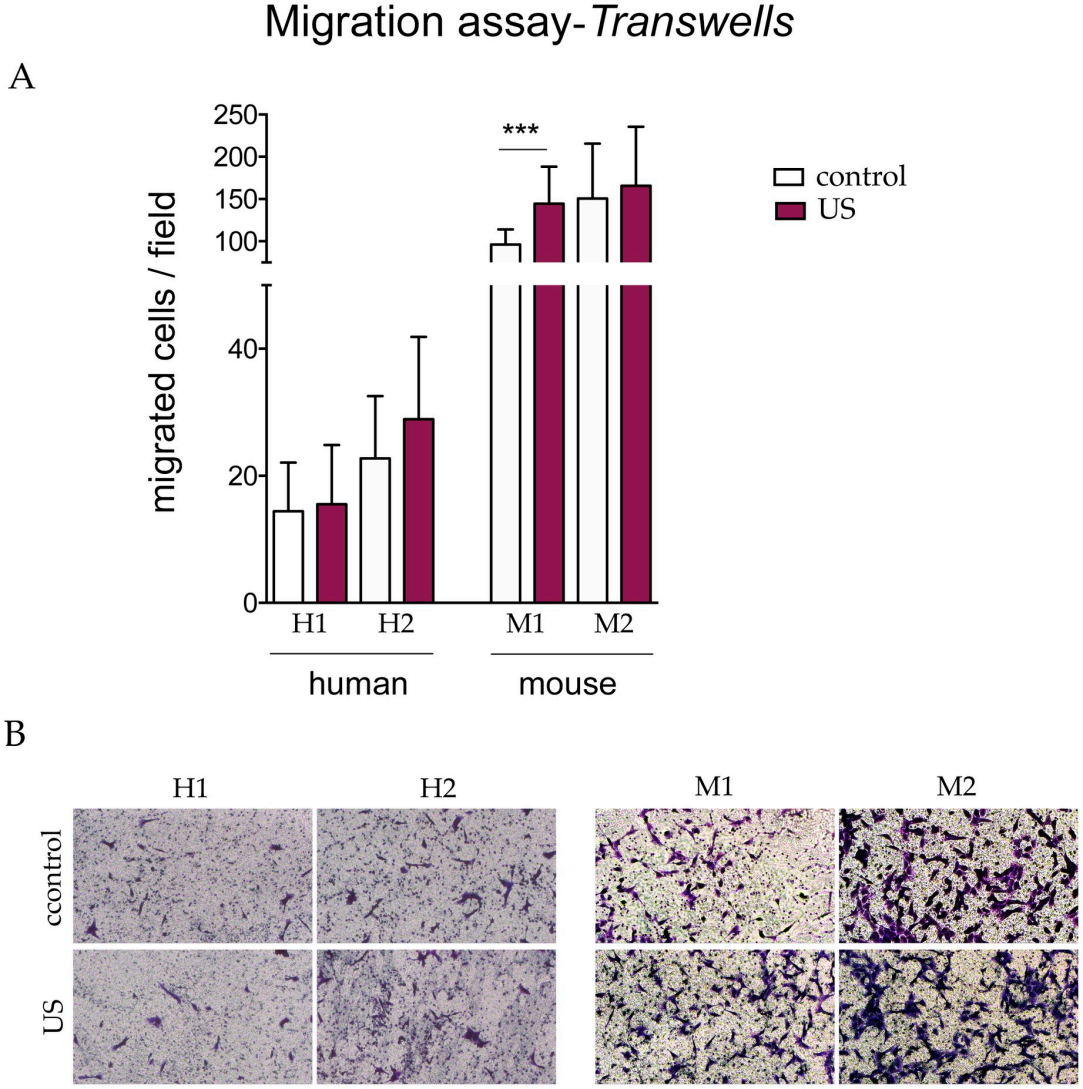
Transwell migration assay. (A) Data are shown from a representative experiment out of three performed and denote mean ± SD. Statistical analysis was performed using Student’s t test. ***p < 0.001. (B) Representative images (10×) of the migrated cells. Shown are two independent samples of mouse (M1 and M2) and human (H1 and H2) s-MPs under control conditions or treated with ultrasound (US).

We also performed a migration assay using Transwell chambers, which were used to assess individual migration through a porous membrane. Of note murine s-MPs had a considerably greater migratory capacity than human counterparts (Fig 6). Similar to the results of the scratch assay, however, no significant changes in migration were observed between human control and LIPUS-stimulated s-MPs (Fig 6). With respect to murine s-MPs, again migration occurred faster in LIPUS-stimulated cells than in control cells, and this was significant for one of the two independent s-MP populations (Fig 6).

**Fig 7:**
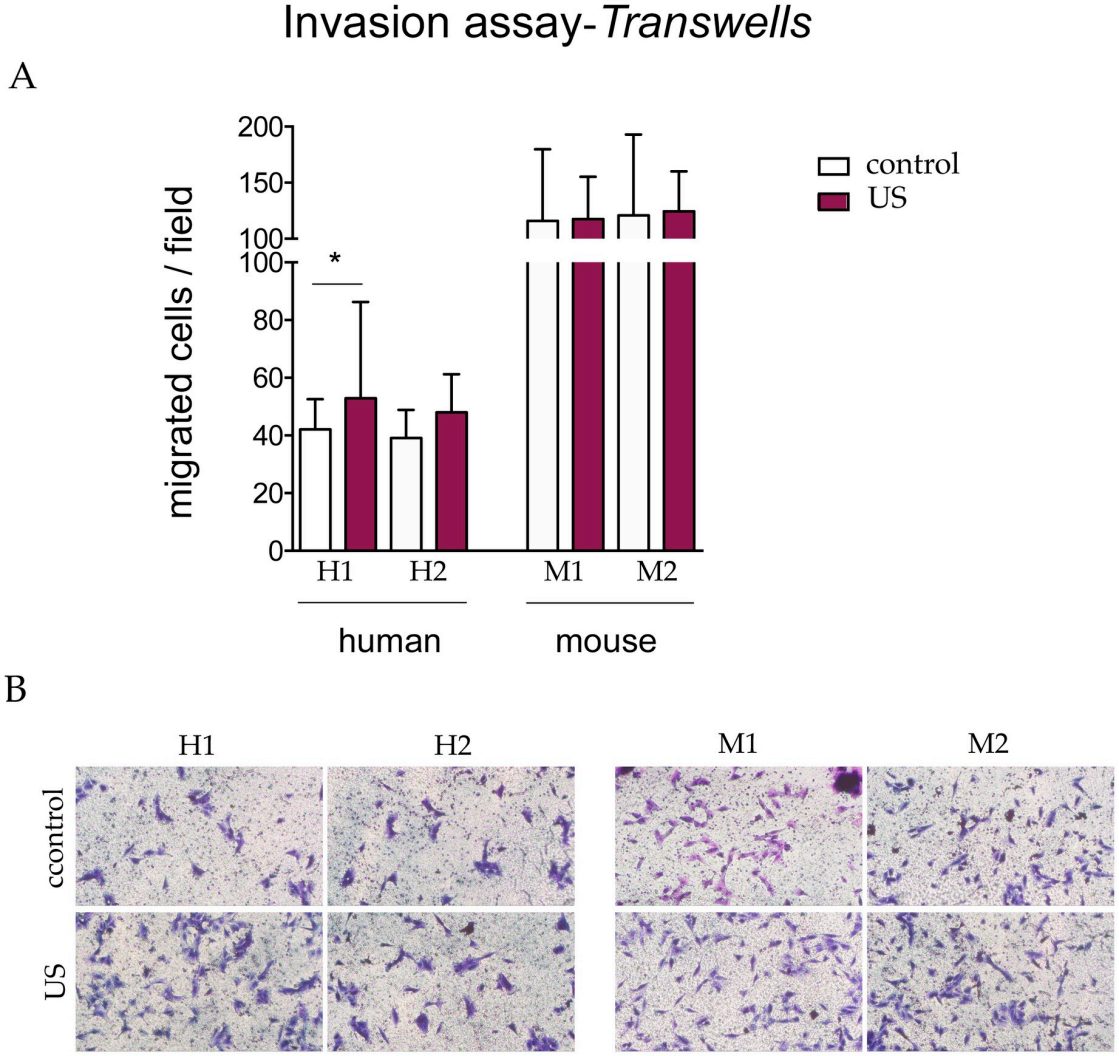
Transwell invasion assay. (A) Data are shown from a representative experiment out of three performed and denote mean ± SD. Statistical analysis was performed using Student’s t test. *p < 0.05. (B) Representative images (10×) of the migrated cells. Shown are two independent samples of mouse (M1 and M2) and human (H1 and H2) s-MPs under control conditions or treated with ultrasound (US).

We repeated the Transwell migration assays using membranes coated with 1% gelatin to create a three-dimensional matrix to assess invasion. Similar to the standard Transwell assay, we observed that murine s-MPs migrated faster than human counterparts (Fig 7). However, there were no differences in the invasion capacity between control and stimulated cells, with the exception of one of the human s-MP samples, in which migration was modestly but significantly greater.

Overall, the results of the migration assays strongly suggest that, at the parameters used, ultrasound stimulation does not improve the migration capacity of s-MPs.

### Low-intensity ultrasound does not modify the expression of ROCK1, COL1A1, TGFB1, LAMB1 and ITGB1

**Fig 8:**
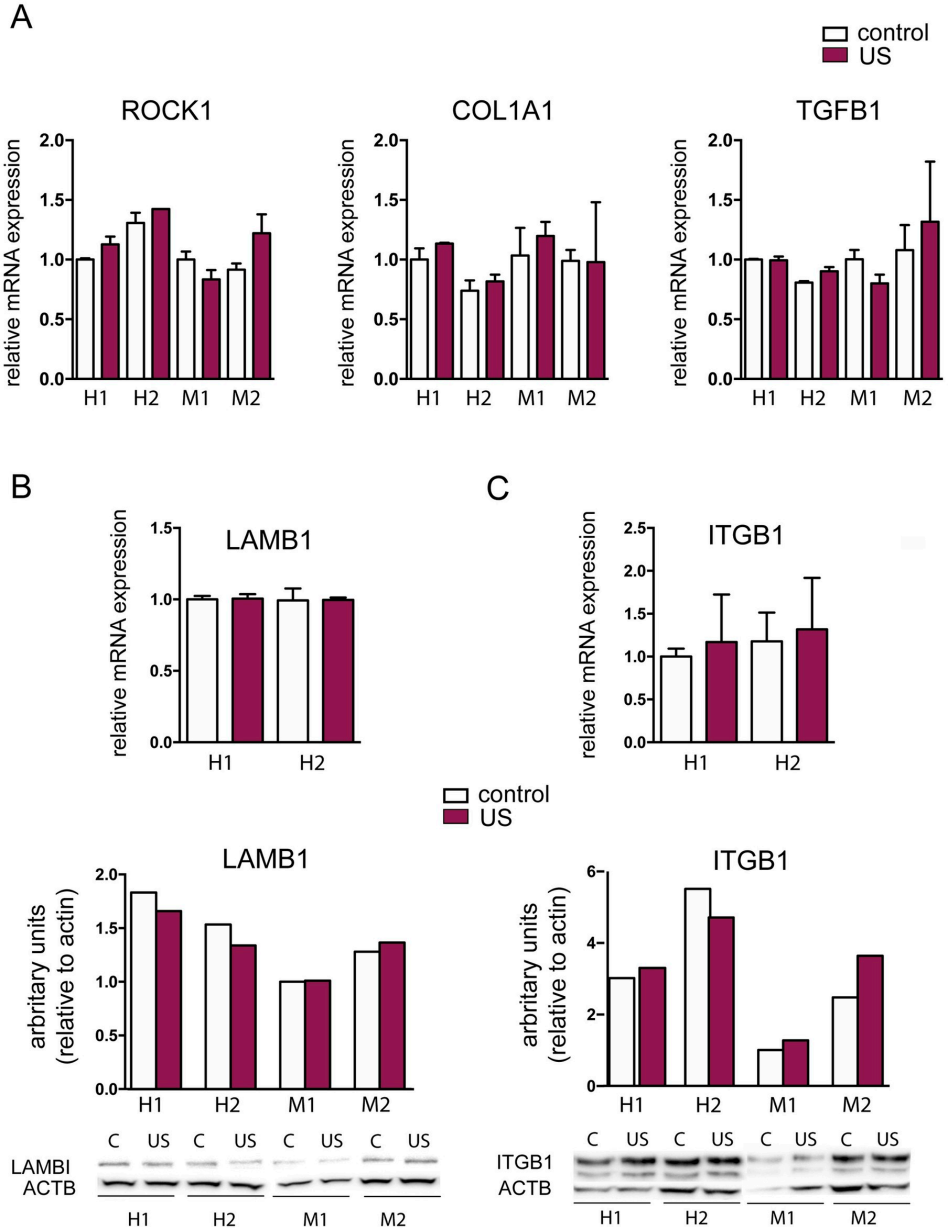
Gene and protein expression analysis. (A) Gene expression of *ROCK1*, *COL1A1*, *TGFB1*. Data are shown from a representative experiment out of three performed and denote mean ± SD. (B) Gene and protein expression of *LAMB1*, and *ITGB1*. Data are shown from a representative experiment out of three performed and denote mean ± SD. Statistical analysis was performed using one-way ANOVA with Bonferroni’s multiple comparisons test. Shown are two independent samples of mouse (M1 and M2) and human (H1 and H2) s-MPs under control conditions or treated with ultrasound (US).

We next analyzed the expression status of several molecules related to mechanotransduction process and to the molecular mechanism of action of LIPUS: ITGB1 (integrin β1), a cellular receptor involved in mechanotransduction; ROCK1 and TGFB1 (transforming growth factor β1), important for cell signaling and function; and LAMB1 (laminin β1) and COL1A1 (type I collagen), important as extracellular matrix components [18,34–37]. RNA and proteins were extracted 5 days after the ultrasound application, and were used for qPCR and western blotting analysis, respectively. Results showed that the mRNA expression of *ROCK1*, *COL1A1*, *TGFB1*, *LAMB*, and *ITGB1* remained unchanged after the ultrasound treatment, both in murine and human s-MPs (Fig 8). Nor were changes detected in the expression of the genes that code for cytokines or one of their receptors: *CXCL12*, *CCL2* and *CXCR4* (S2 Fig).

### RNA-seq

**Fig 9:**
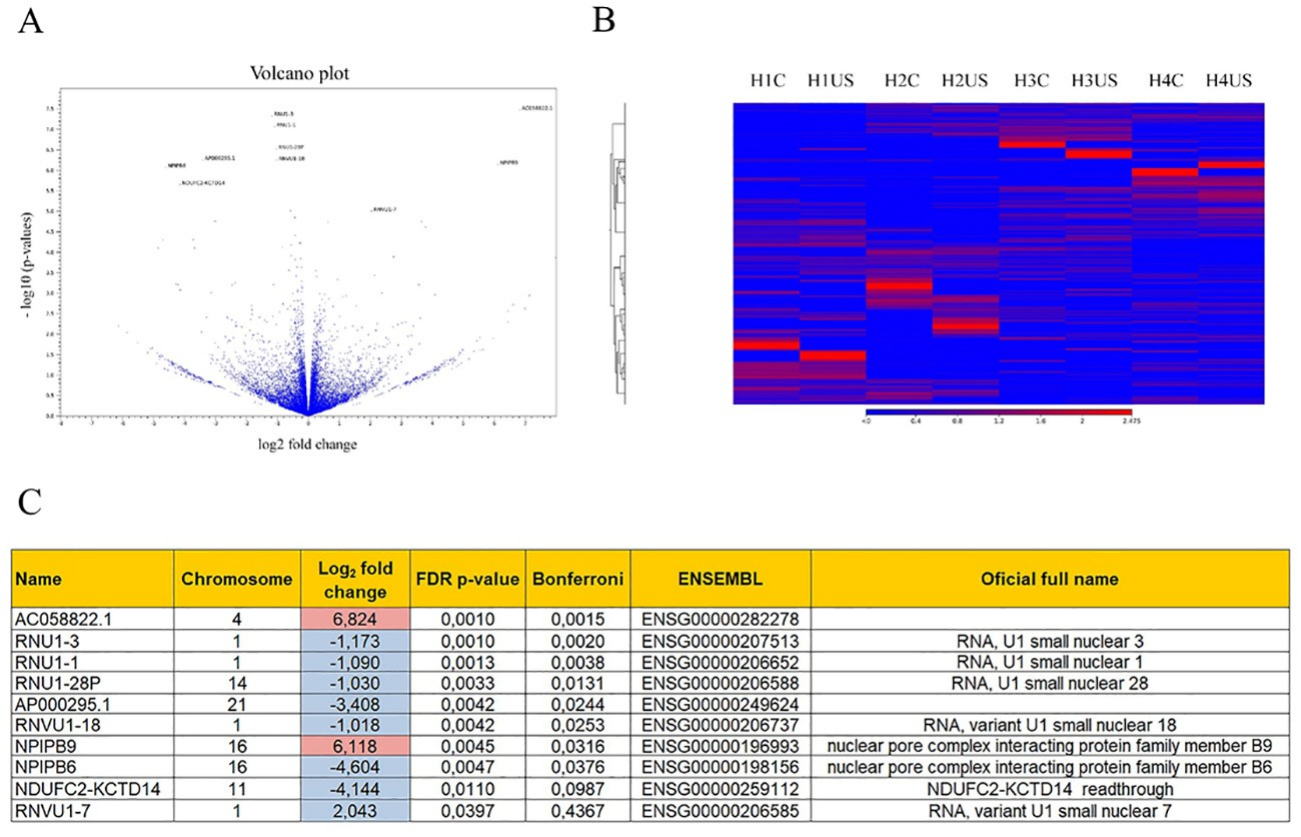
RNA-seq results. (A) Volcano plot showing the 10 genes differentially expressed between control and ultrasound-stimulated (US) human s-MPs. (B) Heat map graph, where the red zones are correlate with upregulated genes and the blue zones with the absence of changes in expression. (C) Differentially expressed genes that fulfill the conditions to present a p-value under 0.05 and fold change over 2 or under -2.

Given the above negative results for the comparison of gene/protein changes with respect to ultrasound application, we performed a more in-depth analysis of gene expression by RNA-seq. Analysis was performed with four different samples of human cells (controls *versus* ultrasound). Surprisingly, but at the same time consistent with previous results, only 10 genes were differentially expressed by the ultrasound protocol, as shown by Volcano plot (Fig 9A).

Heat map analysis showed no overall change in expression patterns (Fig 9B). Of the 10 genes differentially expressed, 2 were upregulated by ultrasound and 8 were downregulated (Fig 9C). Interestingly, among the genes with an altered expression pattern, 5 genes encode for small nuclear RNAs (snRNAs) and 2 genes encode for proteins belonging to the nuclear pore complex.

## Discussion

Ultrasound has proven to have a range of biomedical and other applications. Focusing on the application of LIPUS in wound healing [30,38], by triggering the proliferation and migration of fibroblasts and the concomittant synthesis and deposition of extracellular matrix components, we sought to assess whether low-intensity ultrasound delivered by a conventional device could replicate the positive effects of LIPUS. The benefits of using conventional ultrasound devices are that they are approved by the European Union (for use in Europe) and are found both in hospitals and in physiotherapy clinics, thus negating the need to purchase specific LIPUS devices. Conventional ultrasound devices allow the adjustment of the ultrasound parameters to those within the range of LIPUS, and we used the lowest available intensity, 100 mW/cm^2^ (from intensities up to 3000 mW/cm^2^) in the present study. While the standard parameters of LIPUS are well established (power intensity of 30 mW/cm^2^, frequency of 1.5 MHz, 20% duty cycle), as they are the most often used, there are multiple references to studies with small variations of these parameters, although remaining pulsed ultrasound characterized by its low intensity. Several studies using intensities greater than 30 mW/cm^2^ have reported beneficial results in different settings. In rats, the use of LIPUS at 100 mW/cm^2^ accelerates fracture repair [22]. Likewise, an intensity of 83 mW/cm^2^ induced cardiac differentiation and increased the malleability and mobility of cardiac mesoangioblasts [18]. Much greater intensities (100–1500 mW/cm^2^) have been used on induced pluripotent stem cell-derived neural crest stem cells, which provoked an increase in cell viability, proliferation and neural differentiation [39]. It has been reported that an increase in the time of treatment increases the effectiveness of LIPUS, in a dose-dependent manner [40]. Furthermore, numerous studies have used frequencies other than 1.5 MHz, of which the more typical is 1 MHz [41–43] or 3 MHz [16], both of which are available from conventical therapeutic devices. Closer to our present study, LIPUS intensities of 160/240 mW/cm^2^ and frequency of 3 MHz were used successfully to promote cell proliferation and wound closure in epithelial cells [44]. Given this evidence, we stimulated mesenchymal precursors from mouse and human skin with ultrasound at 100 mW/cm^2^, 3 MHz, 20% duty-cycle for 20 min for three consecutive days, and cell migration and other properties were analyzed. Gross morphological analysis of stimulated s-MPs indicated that the protocol was not detrimental, as morphology was similar to control cells throughout the three-day application. However, it has also been described that LIPUS can induce apoptosis when higher intensities are applied [3,45,46] as occurs when we used 1 W/cm^2^ intensity as a positive control for ultrasound stimulation. Therefore, LIPUS could be a potential tool for the treatment of some cancers [47].

Ultrasound is a mechanical stimulus that is transmitted to the cell by mechanotransduction process. This occurs through different mechanoreceptors (transmembrane proteins) that can be integrins, stretch-activated ion channels (piezo mechanosensitive ion channels) [48–50] which act as mediators between the cytoskeleton and the extracellular matrix [51]. Because ultrasound is known to reorganize the cellular cytoskeleton [18,52], we examined the morphology and cytoskeleton of stimulated s-MPs, particularly actin filaments and microtubules, finding no changes in their distribution.

We next examined cellular proliferation capacity, as this has been shown to be affected by ultrasound stimulation in some studies [15,16]. However, no differences were found between unstimulated and ultrasound-stimulated cells.

To investigate whether the ultrasound protocol could improve cellular migration ability, as previously reported [17,18], and which is a key attribute for enhanced therapeutic potential, we used three complementary assays to examine different types of migration. Overall, none of the three assays showed changes in the migration capacity of s-MPs after ultrasound stimulation, although some modest, but significant, changes were occasionally observed. Of note, however, murine s-MPS had a greater capacity to migrate in Transwell assays than their human counterparts.

Consistent with the previous results, an analysis of different genes and proteins whose expression has been previously linked to proliferation (TGFB1), cytoskeletal reorganization (ITGB1) and migration (ROCK) induced by ultrasound stimulation [18,35,53] revealed no changes in their expression.

Finally, the RNA-seq analysis showed that the ultrasound protocol used in human s-MPs triggered very minor changes in gene expression, and only 10 genes were affected by the treatment. Interestingly, among these differentially expressed genes, 5 genes encode for snRNAs and 2 genes for proteins belonging to the nuclear pore complex. snRNA biogenesis is linked to specialized nuclear suborganelles termed Cajal bodies [54]. snRNAs are involved in the formation and function of the spliceosome [55] and Cajal bodies also act as processing centers for ribonucleoprotein assembly, ribosome biogenesis and telomere maintenance [56]. Variations in the proteins associated with Cajal bodies have been reported following changes in integrins upon mechanical stimuli, indicating that forces on the cellular surface can be transmitted to the nucleus *via* cytoskeletal components [54,57]. Integrins and cadherins are physically coupled to the cellular cytoskeleton, inducing transmission of signals along the proteins. Further, F-actin filaments are joined to microtubules and intermediate filaments, which also connect with nuclear pore complexes, offering an explanation for the relationship between nuclear pores and the cytoskeleton [58]. Our results showing changes in the expression of snRNAs and in proteins of the nuclear pore complex could be a good indicator that ultrasounds are reaching the s-MPs including the nucleus.

While it would appear that ultrasound is safe for use in MPs, which is encouraging, the parameters used in the present study were not optimal to activate certain signaling routes. Of all the parameters that characterize ultrasound waves, intensity is the most relevant, which represents the passage of energy in a given area. The time and duty cycle that we used are standard, whereas the changes introduced were for frequency and intensity. Frequency indicates the number of times a particle experiencing a complete compression and rarefaction cycle in one second and is related to the capacity of the waves to penetrate a body or surface [59]. The use of frequencies of 1.5 MHz or 3 MHz both result in an increase of bone remodeling in rats, with no significant differences between them [60]. However, there is evidence to suggest that 100 mW/cm^2^ might not be the most appropriate intensity to apply to cells. For example, osteoclast activity decreased significantly when 100 mW/cm^2^ was employed instead of 30 mW/cm^2^ [61]. Also, the use of 150 mW/cm^2^ did not further enhance fracture healing in rats when compared with 30 mW/cm^2^ [62], and settings of 30 and 120 mW/cm^2^ in murine osteoblastic cells had different effects on mineralization processes in vitro [63]. In contrast to power settings above 30 mW/cm^2^, intensity values below this can still promote osteogenesis [64]. These findings might explain our results and indicate that at the level of cell stimulation, the use of traditional conventional devices with intensities of 100 mW/cm^2^ would not be the most appropriate conditions, at least in our cell population.

## Limitations and further studies

In our study, for ultrasound parameters selection we have the inherent limitation of the chosen device (physiotherapy ultrasound equipment). We are not able to assess intensities bellow 100mW/cm^2^ and frequencies of 2 or 4 MHz. The use of ultrasound has great potential for several fields of biomedicine. However, it is of great importance to investigate and properly define the specific parameters that control cell fate. Thus, it would be convenient to analyze a range of intensities-frequencies and perform RNA-seq and protein from the day after the stimulation and check the time evolution to improve the knowledge of the stimulation process and how it affects the cells trough the time. It would also be interesting to study the effect of ultrasound on 3D or spheroid-cultured cells in order to better simulate tissue organization in organisms. Although ultrasound is a promising field, much more research is needed to advance in its translation into the clinical practice.

## Conclusions

The final aim of the study was to evaluate the possibilities of physiotherapy ultrasound equipment for skin regeneration. Ultrasound stimulation of s-MPs with this device had no detrimental effects on cell viability; however, the functional properties of the cells studied did not improve, although we believe that the cells received and responded to the ultrasound signal due to the induction of snRNAs and proteins of the nuclear pore complex that are the result of the mechanotransduction process to the nucleus. In conclusion, the use of physiotherapy equipment with LIPUS parameters fails to improve skin precursors capacities and evidence the importance of standardizing ultrasound application parameters and methods.

## Supporting information

S1 Fig 1 W/cm^2^ ultrasound application. Representatives phase contrast images of human and mice s-MPs showing the effects of the stimulation using 1 W/cm^2^ of intensity.
(TIF)

S2 Fig Gene expression level of *CXCL12*, *CCL2* and *CXCR4*. Graph that represent gene expression of *CXCL12*, *CCL2* and *CXCR4*. Similar results were obtained for protein expression analysis of laminin β1 and integrin β1 by western blotting (Fig 8). Thus, the ultrasound application using the selected parameters does not trigger modifications in the expression of LIPUS-related mechanostransduction molecules in s-MPs.
(TIF)

S1 Raw Images (PDF)
