# Supplementary figures and images for: Application of low-intensity pulsed therapeutic ultrasound on mesenchymal precursors does not affect their cell properties
Source: PLoS One. 2021 Feb 11;16(2):e0246261. doi: 10.1371/journal.pone.0246261 (PMC7877602; doi:10.1371/journal.pone.0246261)

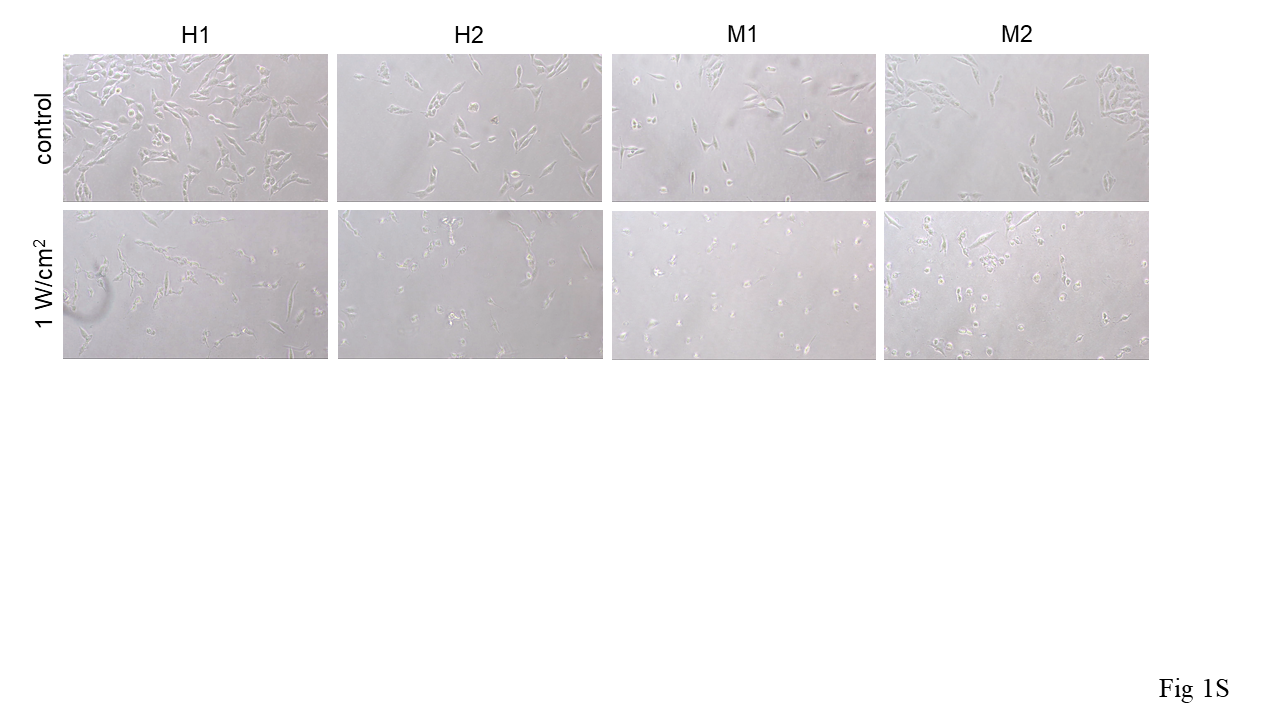

Supplement: S1 Fig — Representatives phase contrast images of human and mice s-MPs showing the effects of the stimulation using 1 W/cm2 of intensity. (TIF) [file pone.0246261.s001.tif]

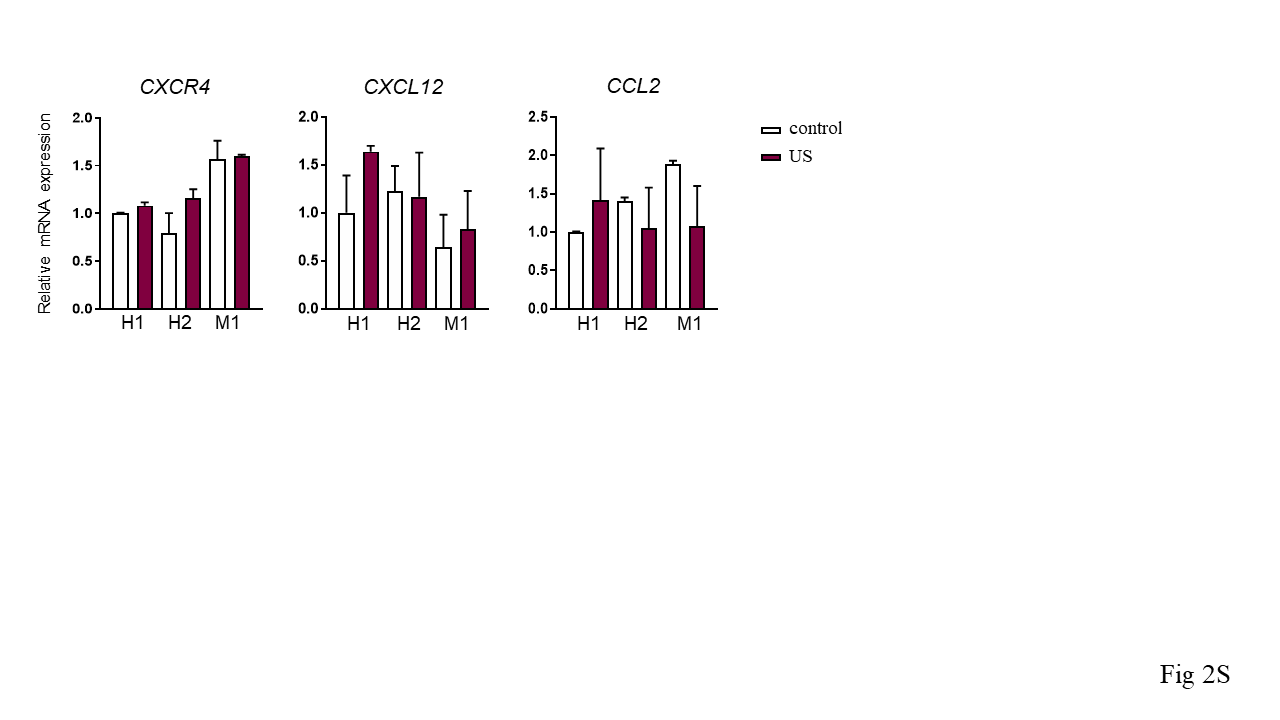

Supplement: S2 Fig — Graph that represent gene expression of CXCL12, CCL2 and CXCR4. Similar results were obtained for protein expression analysis of laminin β1 and integrin β1 by western blotting (Fig 8). Thus, the ultrasound application using the selected parameters does not trigger modifications in the expression of LIPUS-related mechanostransduction molecules in s-MPs. (TIF) [file pone.0246261.s002.tif]
