# Supplementary material for: Application of low-intensity pulsed therapeutic ultrasound on mesenchymal precursors does not affect their cell properties
Source: PLoS One. 2021 Feb 11;16(2):e0246261. doi: 10.1371/journal.pone.0246261 (PMC7877602; doi:10.1371/journal.pone.0246261)
Supplement: S1 Raw Images — (PDF) [file pone.0246261.s003.pdf]

LAMININ WB (used in Figure 8 B)

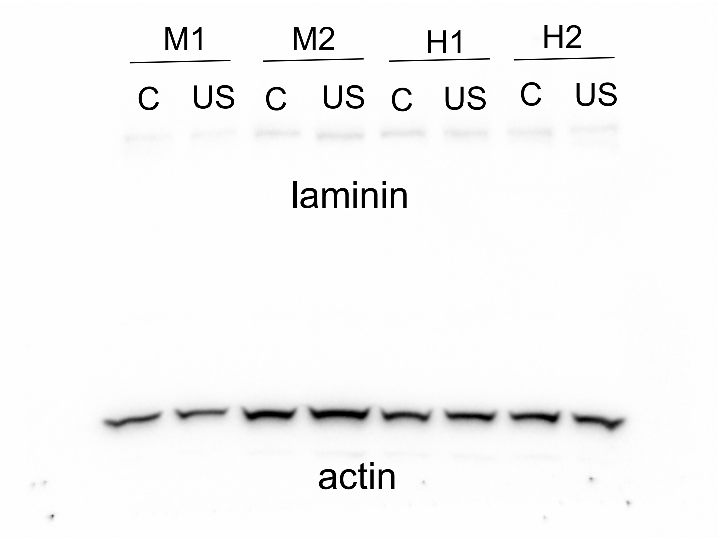

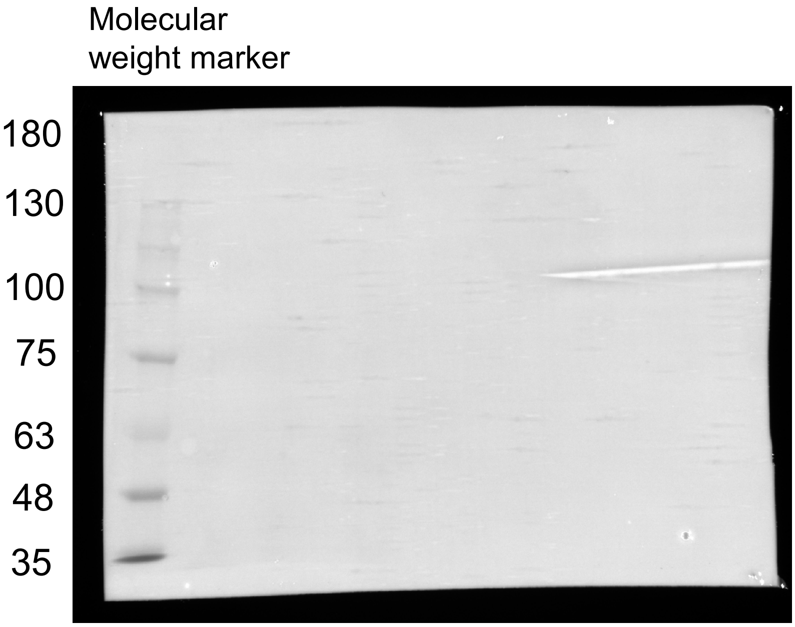

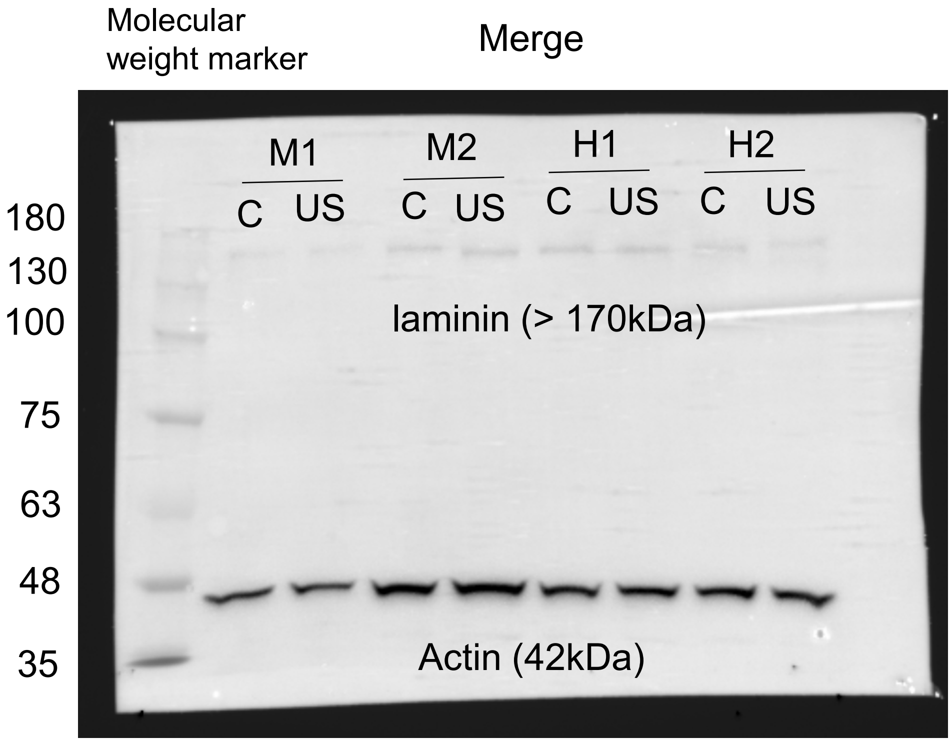

Images adquired with Bio-Rad ChemiDoc™

Samples: Mouse 1 (M1), Mouse 2 (M2), Human 1 (H1) and Human 2 (H2) under control conditions (C) or treated with ultrasound (US).

INTEGRIN β1 WB (used in Figure 8 C)

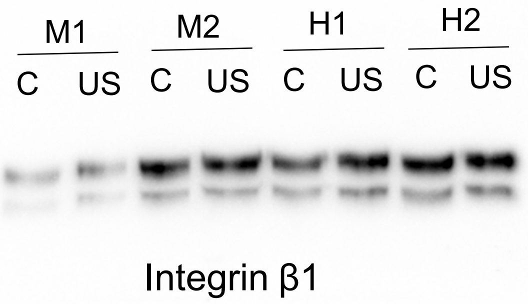

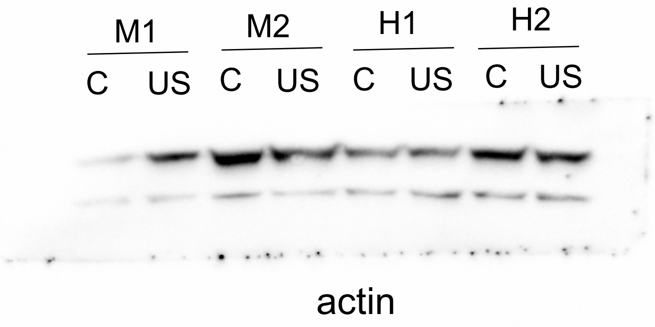

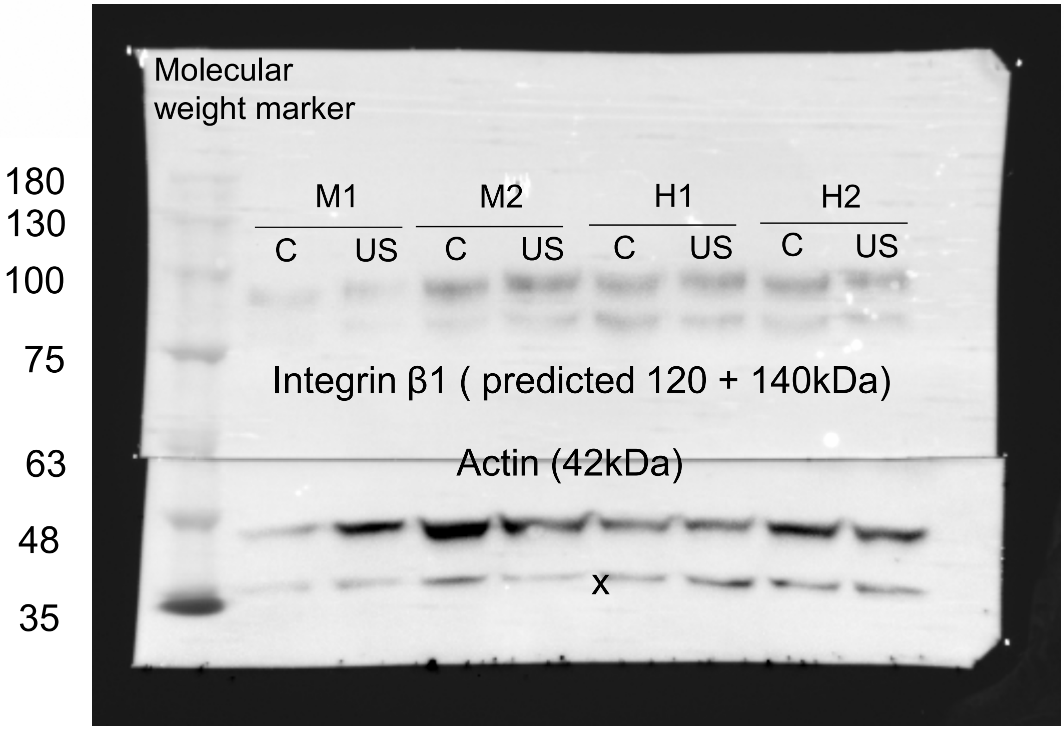

Images adquired with Bio-Rad ChemiDoc™

Samples: Mouse 1 (M1), Mouse 2 (M2), Human 1 (H1) and Human 2 (H2) under control conditions (C) or treated with ultrasound (US).
